# Supplementary material for: Modeling glioblastoma heterogeneity as a dynamic network of cell states
Source: Mol Syst Biol. 2021 Sep 16;17(9):e10105. doi: 10.15252/msb.202010105 (PMC8444284; doi:10.15252/msb.202010105)
Supplement: Supplementary file 5 — Source Data for Figure 3 [file MSB-17-e10105-s001.zip › Figure3A_sourcedata/GSEA_3065/hallmarks_state4.GseaPreranked.1623416296068/HALLMARK_BILE_ACID_METABOLISM.html]

Details for gene set HALLMARK\_BILE\_ACID\_METABOLISM[GSEA]

|  || Dataset | state4 |
| Phenotype | NoPhenotypeAvailable |
| Upregulated in class | na\_pos |
| GeneSet | HALLMARK\_BILE\_ACID\_METABOLISM |
| Enrichment Score (ES) | 0.29786962 |
| Normalized Enrichment Score (NES) | 1.0393516 |
| Nominal p-value | 0.35576922 |
| FDR q-value | 0.40693134 |
| FWER p-Value | 0.997 |
Table: GSEA Results Summary

  

Fig 1: Enrichment plot: HALLMARK\_BILE\_ACID\_METABOLISM      
 Profile of the Running ES Score & Positions of GeneSet Members on the Rank Ordered List

  

| PROBE | GENE SYMBOL | GENE\_TITLE | RANK IN GENE LIST | RANK METRIC SCORE | RUNNING ES | CORE ENRICHMENT || 1 | SCP2 |  |  | 83 | 0.261 | 0.0799 | Yes |
| 2 | PRDX5 |  |  | 123 | 0.237 | 0.1583 | Yes |
| 3 | SOD1 |  |  | 396 | 0.152 | 0.1690 | Yes |
| 4 | IDH2 |  |  | 615 | 0.124 | 0.1781 | Yes |
| 5 | PNPLA8 |  |  | 686 | 0.116 | 0.2082 | Yes |
| 6 | BBOX1 |  |  | 823 | 0.102 | 0.2228 | Yes |
| 7 | PFKM |  |  | 825 | 0.102 | 0.2590 | Yes |
| 8 | PHYH |  |  | 865 | 0.098 | 0.2879 | Yes |
| 9 | RBP1 |  |  | 1171 | 0.078 | 0.2666 | Yes |
| 10 | CAT |  |  | 1179 | 0.077 | 0.2931 | Yes |
| 11 | GSTK1 |  |  | 1381 | 0.067 | 0.2847 | Yes |
| 12 | AR |  |  | 1470 | 0.063 | 0.2930 | Yes |
| 13 | ABCA1 |  |  | 1654 | 0.057 | 0.2838 | Yes |
| 14 | IDH1 |  |  | 1691 | 0.055 | 0.2979 | Yes |
| 15 | HSD17B4 |  |  | 2007 | 0.047 | 0.2639 | No |
| 16 | OPTN |  |  | 2234 | 0.041 | 0.2421 | No |
| 17 | ATXN1 |  |  | 2416 | 0.037 | 0.2262 | No |
| 18 | PEX19 |  |  | 2436 | 0.036 | 0.2361 | No |
| 19 | IDI1 |  |  | 2749 | 0.030 | 0.1965 | No |
| 20 | SLC27A5 |  |  | 2796 | 0.029 | 0.1993 | No |
| 21 | ABCD3 |  |  | 2800 | 0.029 | 0.2090 | No |
| 22 | RXRA |  |  | 3145 | 0.022 | 0.1616 | No |
| 23 | ABCA2 |  |  | 3231 | 0.020 | 0.1552 | No |
| 24 | PEX16 |  |  | 3289 | 0.019 | 0.1526 | No |
| 25 | ABCA3 |  |  | 3335 | 0.017 | 0.1515 | No |
| 26 | PECR |  |  | 3363 | 0.016 | 0.1530 | No |
| 27 | RETSAT |  |  | 3588 | 0.009 | 0.1204 | No |
| 28 | CYP46A1 |  |  | 3740 | 0.004 | 0.0974 | No |
| 29 | MLYCD |  |  | 3850 | -0.002 | 0.0804 | No |
| 30 | SLC23A2 |  |  | 3927 | -0.006 | 0.0702 | No |
| 31 | LONP2 |  |  | 4083 | -0.014 | 0.0503 | No |
| 32 | CROT |  |  | 4120 | -0.017 | 0.0505 | No |
| 33 | ABCA5 |  |  | 4189 | -0.021 | 0.0472 | No |
| 34 | EFHC1 |  |  | 4469 | -0.042 | 0.0173 | No |
| 35 | ISOC1 |  |  | 4505 | -0.045 | 0.0276 | No |
| 36 | ACSL1 |  |  | 4583 | -0.050 | 0.0331 | No |
| 37 | NEDD4 |  |  | 4588 | -0.050 | 0.0504 | No |
| 38 | SLC29A1 |  |  | 4690 | -0.059 | 0.0552 | No |
| 39 | HSD17B11 |  |  | 5042 | -0.086 | 0.0296 | No |
| 40 | FADS1 |  |  | 5324 | -0.111 | 0.0242 | No |
| 41 | FADS2 |  |  | 5544 | -0.135 | 0.0373 | No |
| 42 | DHCR24 |  |  | 5943 | -0.219 | 0.0514 | No |
Table: GSEA details [plain text format]

  

Fig 2: HALLMARK\_BILE\_ACID\_METABOLISM: Random ES distribution      
 Gene set null distribution of ES for **HALLMARK\_BILE\_ACID\_METABOLISM**
